# Thermodynamic and kinetic stabilization of divanadate in the monovanadate/divanadate equilibrium using a Zn-cyclene derivative: Towards a simple ATP synthase model

**DOI:** 10.3762/bjoc.8.8

**Published:** 2012-01-12

**Authors:** Hanno Sell, Anika Gehl, Frank D Sönnichsen, Rainer Herges

**Affiliations:** 1 Otto-Diels Institut für Organische Chemie, Christian-Albrechts-Universität zu Kiel, Otto-Hahn-Platz 4, 24418 Kiel, Germany

**Keywords:** EXSY NMR, NMR titration, supramolecular chemistry, vanadate condensation, ^51^V NMR, Zn-cyclene

## Abstract

For the condensation of anions such as phosphate and ADP to form ATP and water, nature employs sophisticated supramolecular systems to overcome coulomb repulsion and activation barriers. For an attempt to create a simple, analogous chemical system, the dimerization of vanadate is probably the simplest model. We have investigated Zn-benzylcyclene which favors the dimerization thermodynamically as shown by NMR titration. Moreover, EXSY NMR experiments reveal that the vanadate dimer is also kinetically stabilized with respect to hydrolysis by complexation with Zn-cyclene.

## Introduction

**Figure 1:**
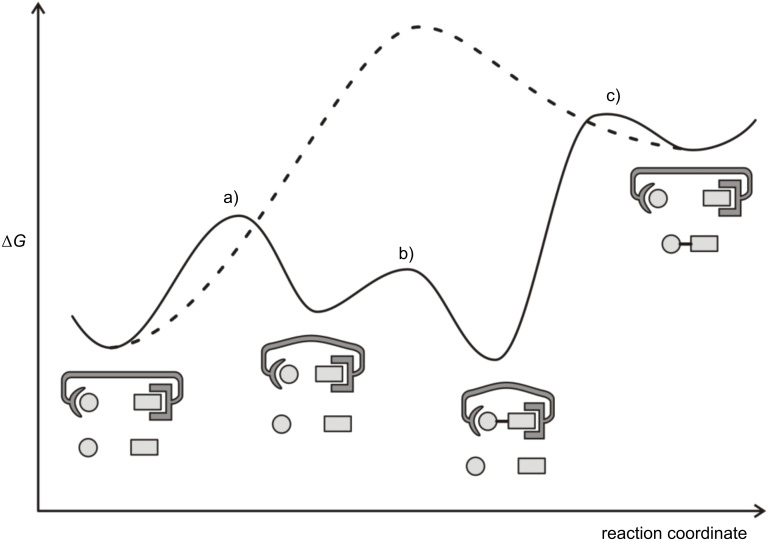
Simplified free energy scheme for driving an endergonic condensation (elimination/addition of water is omitted for simplicity). The non-driven reaction is depicted by a dashed line. a) Moving both complexated anions together, b) bond formation (condensation), and c) product release/reactant binding.

Driving endergonic reactions with external energy sources is one of the challenging and so far unsolved problems in supramolecular chemistry. The best known example in nature is probably the condensation of phosphate and ADP to ATP, driven by a proton gradient across a membrane [1]. There are a number of preconditions that have to be met for such an energy driven condensation of inorganic anions. At least three steps (four stages) are required (Figure 1): a) Moving both complexated anions together, b) bond formation (condensation), and c) product release/reactant binding. In the ATP synthase, the energy is put into the system mainly in step c), thus, avoiding product inhibition [2].

In a first attempt towards the very ambitious goal of creating an artificial ATP synthase model, the a) → b) → c) energy profile has to be carefully designed to operate such a system. Here, we focused on step b). To further simplify the system, we chose the dimerization of vanadate as a model for the phosphate condensation for the following reasons: The condensation of phosphate has a very high activation barrier (disodium phosphate condensates to pyrophosphate at temperatures >250 °C) which is likely difficult to be realized in a prototype artificial system [3]. In contrast to phosphates, mono-, di- and higher vanadates are in equilibrium at room temperature depending on the pH. Moreover, ^51^V is one of the most sensitive NMR nuclei in the periodic table [4]. The concentration of the different vanadate species can be easily determined even in very diluted solutions by integration of the corresponding NMR peaks [5]. Although vanadates are slightly larger than their corresponding phosphates (V–O bond approximately 0.170 nm, P–O bond approximately 0.152 nm) they exhibit a striking similarity in their chemistry [6]. Vanadates are accepted by a number of phosphatase enzymes [7]. Thus, the plethora of information on both natural and artificial phosphate binding systems can be used to design vanadate coordinating ligands. Vice versa, the vanadate model systems will provide insight into the phosphate condensation, e.g., the formation of ATP [8].

A large number of thoroughly investigated enzymes hydrolyze phosphates and provide insight into conceivable mechanisms of phosphate or vanadate condensations. In phosphatases, kinases and ATP synthase, the catalyzed transfer of phosphate usually requires the presence of metal cations such as Zn^2+^, Mg^2+^ and Mn^2+^ in the active site [9]. It is generally assumed that the coordination of the metal cations to the phosphate oxygen atoms reduces the Coulomb repulsion in the transition states or in the intermediates, thus, lowering the activation barrier [10]. In tyrosine phosphatase, this role is taken by hydrogen bonding of the phosphate ion to the positively charged arginine side chain [11]. In the key step of the hydrolysis of pyrophosphate by the yeast phosphatase, two Mg^2+^ ions and arginine H-bonds assist the P–O bond cleavage [12]. A number of artificial enzymes that catalyze phosphate ester hydrolysis have been synthesized and investigated [13]. Kimura et al. demonstrated the acceleration of phosphate ester hydrolysis by Zn^2+^ complexes of triazacyclododecane and tetraazacyclododecane (cyclene) [14]. The efficiency was increased by tethering two or three Zn-cyclene units in an appropriate distance to allow multiple coordination to the phosphate oxygen atoms [15]. Brown et al. were able to catalyze the cyclization (hydrolysis/intramolecular condensation) of 2-hydroxypropyl aryl phosphate using a tethered dimeric Zn-triazacyclododecane [16]. Lehn et al. catalyzed the condensation of acetyl phosphate and phosphate to pyrophosphate using a protonated macrocyclic polyamine [17–19].

## Results and Discussion

### Investigation of the thermodynamic effect

To investigate the fundamental thermodynamic and kinetic effects of metal coordination in inorganic condensation reactions, we focused on the influence of benzyl Zn-tetraazacyclododecane (Zn-benzylcyclene) **1** on the aggregation of vanadate in water.

**Figure 2:**
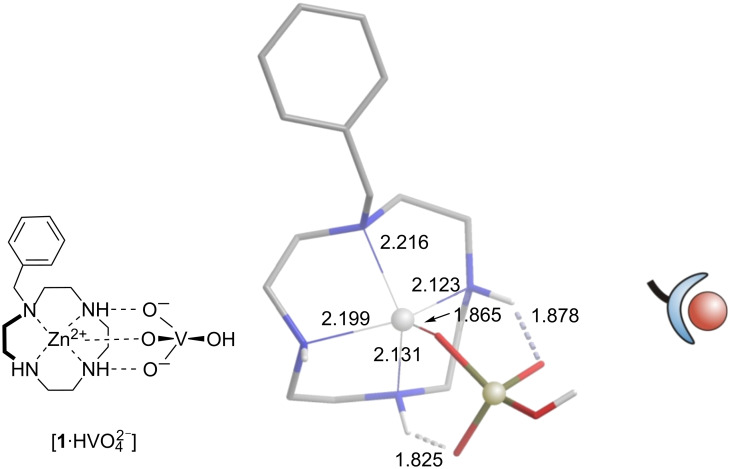
Binding motive of a vanadate zinc benzylcyclene complex (left) as suggested by the results of DFT calculations (B3LYP/6-31G*) (middle). Bond lengths of coordinative bonds and H bridges are given in Å. Metal coordination as well as H–bond formation are operative in binding the oxo-anion to the zinc cyclene unit. The schematic representation (right) is used to improve clarity.

According to DFT calculations (B3LYP/6-31G*) of a number of different geometries, the monovanadate anion is bound to the Zn-cyclene unit by forming one coordination bond to the Zn^2+^ ion and two hydrogen bonds to the NH groups of the cyclene ring (Figure 2). The formation of a bidentate complex (two vanadate oxygens coordinating with Zn^2+^) is considerably less favorable. This is also in agreement with X-ray structures of Zn-cyclene phosphate complexes [15].

**Figure 3:**
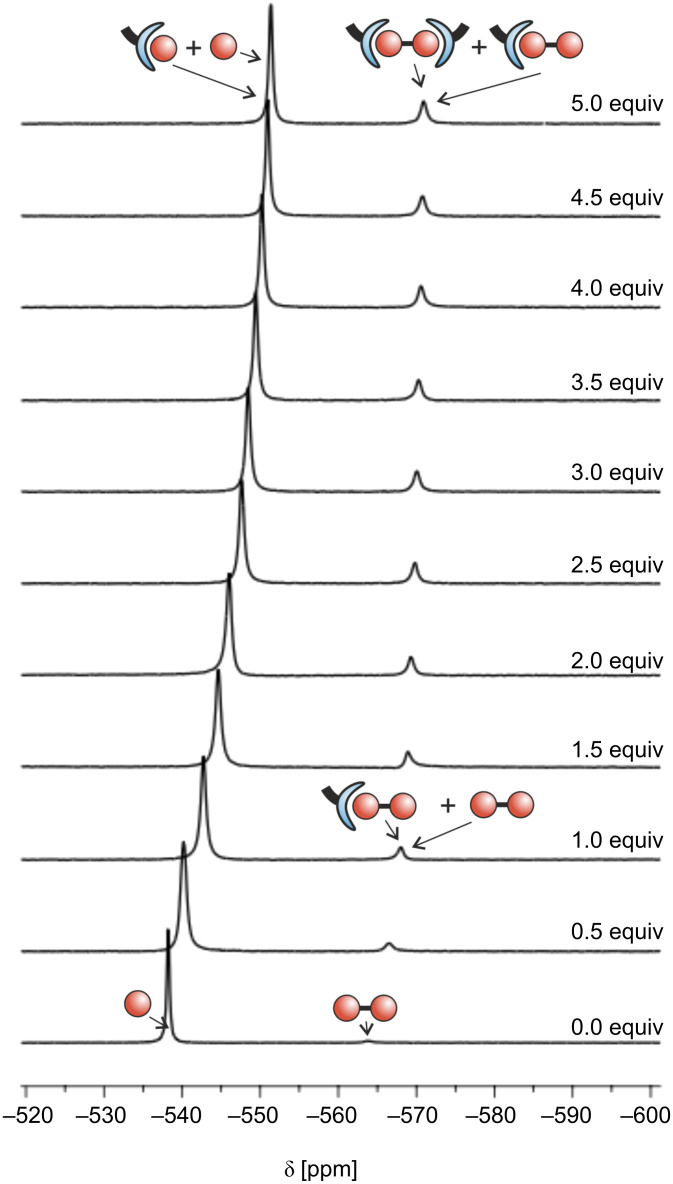
^51^V NMR titration at pH 9.5 ([V]_t_ = 1.5 mM, [**1**]_t_ = 0 to 7.5 mM (0 to 5 equiv), 100 mM CHES). [V]_t_ and [**1**]_t_ are the total molar concentrations of vanadium and Zn-benzylcyclene **1**. The predominant species in equilibrium are represented schematically (Figure 2).

For the analysis of the multicomponent system, 1-D ^51^V NMR titrations of Na_3_VO_4_ with ligand **1** were performed at three different pH values at 25 °C (pH ~9.5 CHES buffer (*N*-cyclohexyl-2-aminoethanesulfonic acid); pH ~8.5 EPPS buffer (3-[4-(2-hydroxyethyl)-1-piperazinyl]propanesulfonic acid); pH ~7.6 HEPES buffer (2-[4-(2-hydroxyethyl)piperazin-1-yl]ethanesulfonic acid)) (Figure 3, see also Supporting Information File 1). These alkaline pH values were chosen because monovanadate is the main species in this range. Like the monovanadate, the di- and tetravanadate exist primarily in the neutral region.

The ^51^V NMR titration at pH 9.5 is shown in Figure 3 (for the corresponding spectra recorded at pH 7.6 and 8.5 see Supporting Information File 1). The addition of Zn-benzylcyclene **1** leads to an upfield shift of the monovanadate and the divanadate signal at all pH values that were investigated. This shift indicates that Zn-benzylcyclene **1** is capable of complexing both mono- and divanadate. The monovanadate signal remains the main signal throughout the titrations, however, the signal of the divanadate increases upon addition of Zn-benzylcyclene. By raising the concentration of benzylcyclene **1**_t_ from 0 to 7.5 mM in a system with a total concentration of vanadium [V]_t_ of 1.5 mM at a pH value of 9.5, the ratio of vanadium bound in divanadate species rises from 5% to 23%, while the ratio of vanadium bound in monovanadate decreases from 95% to 77%. Hence, Zn-benzylcyclene **1** favors the condensation of monovanadate to divanadate.

To determine the stoichiometry and stability of the most important species in this system, we included all vanadate oxo-anions that are known to exist in aqueous solution [20–21] and the corresponding complexes with ligand **1** in different stoichiometries as basis of a complex formation model. This model was refined with respect to the experimental data.

In the approach mentioned above, the equilibria of the multi component system were described mathematically by mass balance equations. When the equilibrium species are written with H^+^, VO_4_^3−^, and Zn-benzylcyclene **1** as components, they are formed according to the general expression (Equation 1) [21]:

[1]
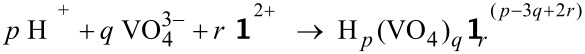


The formation constants of the species *β_pqr_* are defined using the concentrations of **1**, VO_4_^3−^ and of the protons according to Equation 2.

[2]
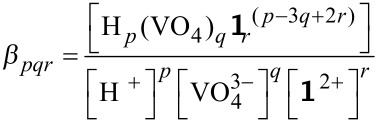


The substitution of the complex species concentration in the mass balances of the components leads to the mass balance equation system (3). The concentrations of the free components are the variables; the stability constants are the parameters to be optimized.

[3]
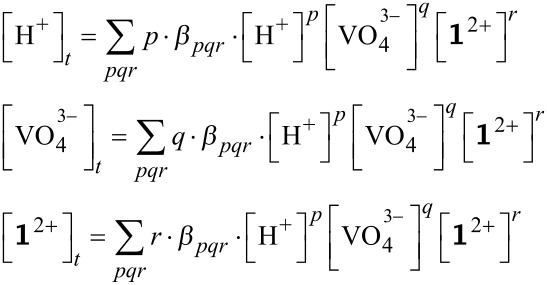


In the ^51^V NMR titrations for the analysis of the vanadate/Zn-benzylcyclene system the total vanadium concentration [V]_t_ at each pH value was 1.5 mM, and the total concentration of **1** ([**1**]_t_) was varied from 0 to 7.5 mM. The NMR data were obtained by careful deconvolution and integration of the signals using routines included in the MestreNova program [22]. The mathematical analysis of the NMR data was performed with the program system LAKE [23] for chemical equilibrium analysis and with MS-Excel [24]. Data of all titrations (78 titration points in total) were included in the calculation. The formation constants for the vanadium oxo-anions of the VO_4_^3−^/H^+^-subsystem [21], the p*K*_a_ value of Zn-benzylcyclene **1** [14] as well as the p*K*_a_ values of the buffers [25] were adopted from literature. The influence of the ionic medium was taken into account in the calculation by means of the Davies equation [26]. The most important ternary species in the equilibrium model that fit the recorded data are presented in Table 1.

**Table 1:** Stoichiometry and formation constants of the complex species in the proton/vanadium/Zn benzylcyclene system.

p, q, r^a^	formula^b^	lg *β*_pqr_^c^ (3σ)

1, 1, 1	HVO_4_**1**	16.7 (0.3)
2, 2, 1	V_2_O_7_**1**^2−^	32.2 (0.1)
2, 2, 2	V_2_O_7_**1**_2_	35.7 (0.8)
3, 2, 1	HV_2_O_7_**1**^−^	40.6 (0.3)
3, 2, 2	HV_2_O_7_**1**_2_^2+^	43.6 (0.4)
8, 4, 2	V_4_O_12_**1**_2_	99.9 (0.7)
8, 4, 4	V_4_O_12_**1**_4_^4+^	105.6 (1.8)

^a^Stoichiometric coefficients according to the formation from the components H^+^, VO_4_^3−^ and **1** (Equation 1); ^b^formula of the oxo-anion complex. The water formed according to the formation reaction from the components is not given; ^c^ionic strength, *I* = 150 mM, *T* = 298 K.

**Figure 4:**
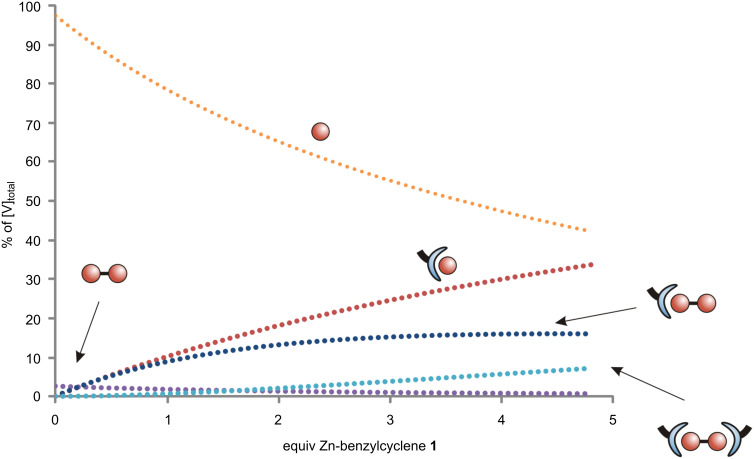
Speciation of vanadium in a solution containing 1.5 mM Na_3_VO_4_, 100 mM CHES (pH = 9.5) and a Zn-benzylcyclene **1** amount varying from 0 to 5 equiv.

According to these data, the association constant for the protonated monovanadate HVO_4_^2−^ with **1** is about 10^3.8^ M^−1^, while the association constant for V_2_O_7_^4−^ is *K*_a_ = 10^6.2^ M^−1^. The p*K*_a_ value for the coordinated divanadate is 8.4 and therewith, 1.9 p*K*_a_ units lower than for the uncoordinated V_2_O_7_^4−^ ion. The binding of a second Zn-benzylcyclene unit proceeds with an association constant of 10^3.5^ M^−1^. Consequently, the affinity of the divanadate for both the proton and the second Zn-benzylcyclene is lowered by the coordination of the first Zn-benzylcyclene unit. A speciation diagram for the titration at pH 9.5 based on these data is given in Figure 4. Fits of calculated and experimental ^51^V NMR integral data at pH 7.6, 8.5, and 9.5 are given in the Supporting Information File 1.

The formation constants given in Table 1 can be used to calculate the free enthalpy (Δ*G*_1_^0^, Δ*G*_2_^0^, Δ*G*_3_^0^) of the condensation reactions (Equations 4–9):


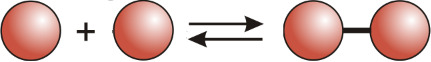


[4]
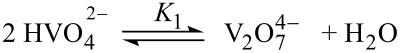


[5]
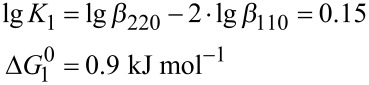



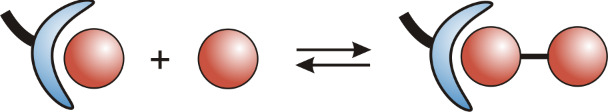


[6]
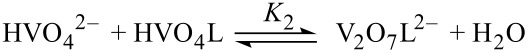


[7]
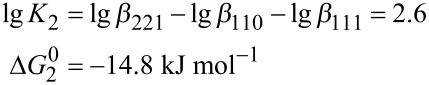



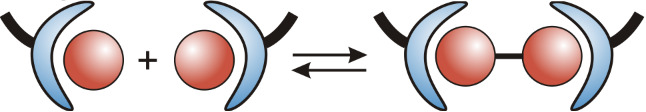


[8]
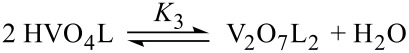


[9]
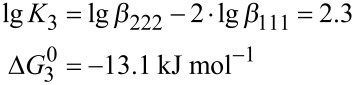


The analysis reveals that the free reaction enthalpy for the condensation of hydrogen monovanadate is lowered by the complexation of the vanadium oxo-anions with Zn- benzylcyclene. In the absence of the ligand, the dimerization of monovanadate is almost thermoneutral (Δ*G*_1_^0^ = 0.9 kJ mol^−1^). However, the condensation of a free monovanadate with a monovanadate bound to Zn-benzylcyclene is exothermic (Δ*G*_2_^0^ = −14.8 kJ mol^−1^) and the reaction of two complexed monovanadates is less exothermic (Δ*G*_3_^0^ = −13.1 kJ mol^−1^). Hence, the condensation is favored by the complexation with Zn-benzylcyclene and one equiv of the ligand is more effective than two.

Further insight into the stabilization of divanadate by Zn-benzylcyclene is obtained by analyzing the ligand exchange reactions (Equation 10 and Equation 11). While the transfer of a ligand from hydrogenmonovanadate to divanadate is exergonic (Δ*G*_10_^0^ = −13.7 kJ mol^−1^), the transfer of a second ligand from another hydrogen monovanadate is endergonic (Δ*G*_12_^0^ = 1.1 kJ mol^−1^).


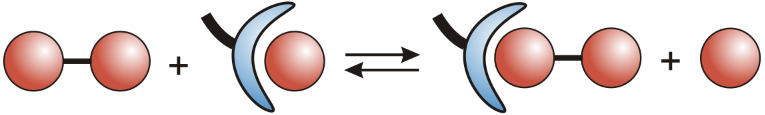


[10]
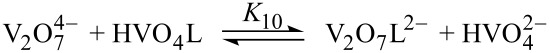


[12]
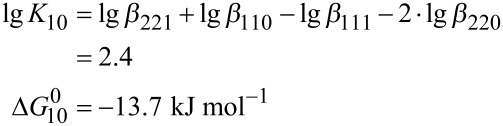



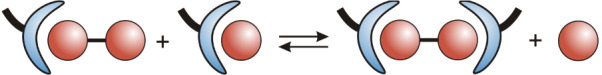


[11]
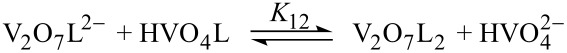


[13]
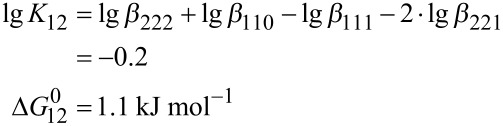


Accordingly, a complexed divanadate is destabilized by the coordination of a second ligand.

### Investigation of the kinetic effect

**Figure 5:**
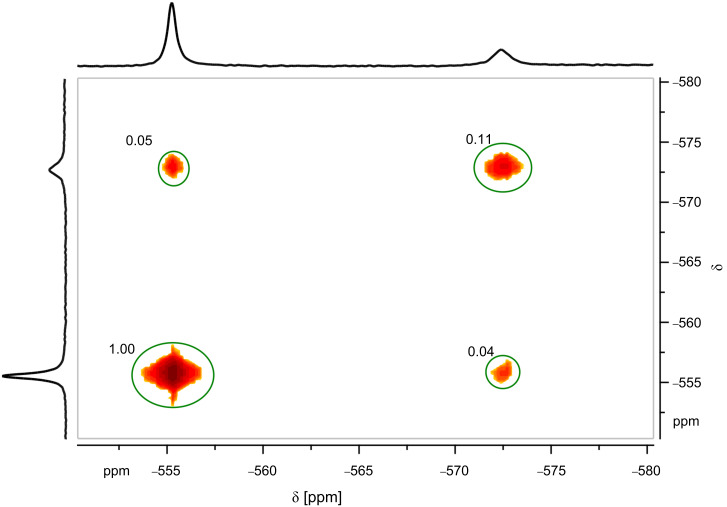
^51^V EXSY NMR spectrum (*t*_mix_ = 1 ms) of a solution containing 1.5 mM Na_3_VO_4_, 3 mM Zn-benzylcyclene and 100 mM EPPS (pH = 7.9).

The influence of the complexation on the rates of condensation and hydrolysis of the involved vanadium oxo-anions was examined by ^51^V EXSY NMR experiments [27–28]. ^51^V EXSY NMR data were collected for solutions buffered with EPPS and HEPES and 1.5 mM total vanadium concentration at 25 °C. For each buffer solution, the amount of Zn-benzylcyclene **1** was varied. The mixing time was 1 ms (Figure 5).

The data show a decrease for the pseudo first order rate constant of the condensation (*k*_12_ in Equation 14) as well as for the hydrolysis (*k*_21_ in Equation 15), while the influence on the rate constant of the hydrolysis is stronger.

[14]
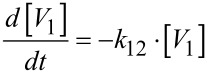


[15]
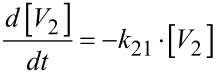


In the HEPES buffered solution (pH 7.6), the rate constant for the condensation decreased from 73 s^−1^ to 68 s^−1^ upon the addition of Zn-benzylcyclene (3 mM), while the rate constant for the hydrolysis decreased from 206 s^−1^ to 89 s^−1^. Compared to that, in the EPPS buffered solution (pH 8.0), the rate constant for condensation decreased from 23 s^−1^ to 14 s^−1^ upon the addition of Zn-benzylcyclene **1** (3 mM), while the rate constant for the hydrolysis decreased from 164 s^−1^ to 70 s^−1^.

The fact that the condensation of monovanadate is slower in the presence of the ligand is probably due to steric reasons. The nucleophilic attack on the complexed monovanadate is only possible from the non-shielded side. Moreover, the role of the monovanadate acting as the electrophile is defined by complexation which reduces the probability of effective collisions and, thus, decreases the entropy of activation. Both effects, obviously, overcompensate the increased reactivity of the vanadate acting as the electrophile which is induced by complexation (decreased Coulomb repulsion of the two negatively charged reactants). The same arguments are true for the hydrolysis of divanadate (attack of water). However, the thermodynamic stabilization of divanadate further increases the barrier of hydrolysis (provided that the transition state is less effected). Therefore, the rate constant of the divanadate hydrolysis is lowered more strongly by complexation than the rate constant for the condensation of monovanadate.

## Conclusion

In conclusion, the condensation of monovanadate to divanadate is considerably favored in the presence of the ligand **1** (Zn-benzylcyclene). In the absence of the ligand at pH 9.5 the condensation is almost thermoneutral (Δ*G*_1_^0^ = 0.9 kJ mol^−1^) whereas upon addition of the ligand the dimerization reaction is exergonic (Δ*G*_2_^0^ = −14.8 kJ mol^−1^). This is mainly due to the fact that the free binding enthalpy of ligand **1** to divanadate is 13.7 kJ mol^−1^ larger than the complex formation with monovanadate.

Complexation with **1** also favors the formation of divanadate kinetically by decreasing its rate of hydrolysis from 164 s^−1^ to 70 s^−1^ upon addition of ligand **1**, as determined by ^51^V EXSY NMR experiments.

Dimerization, however, is less favorable if both reacting monovanadate units are complexed with ligand **1** (Δ*G*_3_^0^ = −13.1 kJ mol^−1^) compared to the reaction of a complexed vanadate with a free monovanadate (Δ*G*_2_^0^ = −14.8 kJ mol^−1^). For the design of an artificial system driving endergonic condensations (Figure 1), we draw the conclusion that only one of the binding sites should provide metal coordination to the vanadate (or phosphate) and the other binding site should associate the nucleophilic anion by neutral hydrogen bonds.

Similar effects obviously hold for natural systems – particularly ATP synthase, phosphatases and kinases catalyzing the condensation, hydrolysis and phosphate transfer in phosphate esters and phosphate oligomers – which are unsymmetric with respect to metal complexation of the substrates. In the P–O bond forming/cleaving step of yeast pyrophosphatase, the phosphate group acting as the electrophile is activated by coordination to Mg^2+^ as well as by hydrogen bonding to a guanidinium group [12]. The electrophilicity of ADP in ATP synthase is even further increased by electron transfer to Mg^2+^ forming a radical ion pair [29]. Similar to the ATP synthase, a simplified artificial system should prevent product inhibition by actively releasing the product. This could be realized by a (photo)switchable ligand, see step a) and c) in Figure 1.

## Experimental

### Synthesis

**1-Benzyl-1,4,7,10-tetraazacyclododecane (benzylcyclene):** Benzylchloride (1.00 mL, 8.69 mmol) and cyclene (6.00 g, 34.8 mmol) were dissolved in dry chloroform (240 mL). Under nitrogen atmosphere, triethylamine (1.45 mL, 10.4 mmol) was added and refluxed for 20 h. After cooling, chloroform (200 mL) was added, and the organic phase was washed three times with 1 N sodium hydroxide solution (200 mL) and three times with distilled water (200 mL). The organic phase was dried over potassium carbonate and the solvent was removed under reduced pressure [30].

For efficient purification, the crude product (2.27 g) was protected with di-*tert*-butyl dicarbonate (3 equiv) in dry dichloromethane (100 mL). After removal of the solvent the product was purified by column chromatography (cyclohexane:ethylacetate = 1:1, *R*_f_ = 0.58). Yield: 4.24 g (87%); ^1^H NMR (500 MHz, CD_2_Cl_2_) δ 7.3–7.2 (m, 5H, C*H*_aryl_), 3.65 (s, 2H, Ar-C*H*_2_), 3.52 (br s, 4H, C*H*_2_), 3.4–3.1 (m, 8H, C*H*_2_), 2.60 (br s, 4H, C*H*_2_), 1.41, 1.36 (2 s, 27H, C*H*_3_) ppm; ^13^C NMR (125 MHz, CD_2_Cl_2_) δ 155.7 (C=O), 137.5 (*C*_aryl,q_), 128.5, 127.6, 123.6, (*C*_aryl_), 80.0, 79.5, 79.4 (*C*(CH_3_)_3_), 57.5 (Ar-*C*H_2_), 50.7, 50.1, 48.2 (*C*H_2_), 28.8, 28.6 (*C*H_3_) ppm.

To release the free amine, the boc-protected benzylcyclene (3.76 g, 6.69 mmol) was dissolved in dry dichloromethane (30 mL). Trifluoroacetic acid (5 mL) was added under a nitrogen atmosphere and the mixture stirred at room temperature for 12 h. Under ice cooling sodium hydroxide (3 g) and distilled water (30 mL) were added. The organic layer was separated, and the aqueous phase extracted five times with 50 mL dichloromethane. The combined organic phases were dried over potassium carbonate and the solvent was removed under reduced pressure to yield a colorless solid (1.52 g, 86%); ^1^H NMR (500 MHz, CD_2_Cl_2_) δ 7.3–7.2 (m, 5H, C*H*_aryl_), 3.64 (s, 2H, Ar-C*H*_2_), 2.8–2.4 (m, 16H, C*H*_2_) ppm; ^13^C NMR (125 MHz, CD_2_Cl_2_) δ 139.9 (*C*_aryl,q_), 129.5, 128.6, 127.4 (*C*_aryl_), 60.1 (Ar-*C*H_2_), 51.8, 48.1, 46.6, 46.1 (*C*H_2_) ppm.

In order to regain the excess of cyclene used in the first step, the aqueous phase was concentrated under reduced pressure and neutralized with 1 N HCl until a white precipitate formed. The precipitate was filtered to yield pure cyclene (2.8 g). ^1^H NMR (200 MHz, DCl_3_) δ 2.65 (s, 16H, C*H*_2_) ppm.

**Zinc-1-benzyl-1,4,7,10-tetraazacyclododecane nitrate (1):** 1-Benzyl-1,4,7,10-tetraazacyclododecane (1.00 g, 3.81 mmol) was dissolved in methanol (20 mL). A zinc nitrate solution in water (49.7 mM, 76.6 mL) was added and the solution was stirred for 1 h at room temperature. The solvent was removed under reduced pressure and the colorless solid was dried. Yield: 100 %; ^1^H NMR (500 MHz, D_2_O) δ 7.4–7.3 (m, 5H, C*H*_aryl_), 3.94 (s, 2H, C*H*_2_), 3.2–3.0 (m, 4H, C*H*_2_), 2.9–3.0 (m, 12H, C*H*_2_) ppm; ^13^C NMR (125 MHz, D_2_O) δ 138.5 (*C*_aryl,q_), 131.3, 129.1, 128.7 (*C*_aryl_), 56.7 (Ar-*C*H_2_), 49.1, 44.5, 43.6, 42.2 (*C*H_2_) ppm; ESI *m*/*z* (%): 363.1 (100), 364.1, 365.1, 366.1, 367.1, 368.1 [C_15_H_26_N_4_Zn(OH_2_)_2_], 326.1 (78), 327.1, 328.1, 329.1, 330.1, 331.1 [C_15_H_25_N_4_Zn].

### NMR experiments

#### NMR titrations

For the NMR titrations three different buffer stock solutions were used: pH 7.6: HEPES (4-(2-hydroxyethyl)piperazine-1-ethanesulfonic acid), pH 8.5: EPPS (4-(2-hydroxyethyl)-1-piperazinepropanesulfonic acid) and pH 9.5: CHES (2-(cyclohexylamino)ethanesulfonic acid). The stock solutions had a total buffer concentration of 100 mM and 50 mM of sodium hydroxide. They were prepared by dissolving the appropriate amount of buffer and sodium hydroxide in 90% deionized water and 10% deuterium oxide. To obtain the vanadate stock solution (50 mM) sodium orthovanadate (92.0 mg) was dissolved in the respective buffer solution (10 mL). For the receptor stock solution (10 mM) the receptor **1** (45.2 mg) was dissolved in 10 mL of the respective buffer solution.

All measurements were carried out at a 1.5 mM total vanadate concentration and a 100 mM buffer concentration. The samples of the ^51^V NMR titrations were prepared by mixing the appropriate amount of the vanadate stock solution and the receptor **1** stock solution in an NMR tube. Finally, the volume of each sample was brought to a volume of 600 µL by addition of the appropriate amount of the respective buffer stock solution. All NMR spectra were recorded on a Bruker FT-NMR-spectrometer DRX 500. Vanadium chemical shifts are reported relative to the external reference VOCl_3_ (0 ppm).

### EXSY measurements

The EXSY measurements were performed at two different pH values (pH 7.6 and pH 8.0) and at a total vanadate concentration of 1.5 mM. The concentration of **1** was 3.0 mM. For all stock solutions deionized water was used as solvent. The samples were prepared in the following way: The buffer stock solutions had a total buffer concentration of 500 mM (HEPES for pH 7.6, EPPS for pH 8.0) and 250 mM of sodium hydroxide. The vanadate stock solution contained of 50 mM sodium orthovanadate, and the concentration of the Zn-benzylcyclene **1** stock solution was 10 mM. For each experiment 60 μL of the vanadate stock solution, 400 μL of the respective buffer stock solution, and 600 μL of the Zn-benzylcyclene **1** stock solution were mixed with 100 μL deuterium oxide and 840 μL deionized water. The NMR tubes were then filled with 600 μL of the mixture. In all experiments a mixing time of 1 ms was applied.

## Supporting Information

File 1 ^51^V NMR spectra and data fitting results.
