# Supplementary material for: Thermodynamic and kinetic stabilization of divanadate in the monovanadate/divanadate equilibrium using a Zn-cyclene derivative: Towards a simple ATP synthase model
Source: Beilstein J Org Chem. 2012 Jan 12;8:81–9. doi: 10.3762/bjoc.8.8 (PMC3302101; doi:10.3762/bjoc.8.8)
Supplement: File 1 — 51V NMR spectra and data fitting results. [file Beilstein_J_Org_Chem-08-81-s001.pdf]

Supporting Information

for

Thermodynamic and kinetic stabilization of divanadate in the monovanadate/divanadate equilibrium using a Zn-cyclene derivative: Towards a simple ATP synthase model

Hanno Sell, Anika Gehl, Frank D. Sönnichsen and Rainer Herges*

Address: Otto-Diels Institut für Organische Chemie, Christian-Albrechts-Universität zu Kiel, Otto-Hahn-Platz 4, 24418 Kiel, Germany

Email: Rainer Herges - rherges@oc.uni-kiel.de

* Corresponding author

^{51}V NMR spectra and data fitting results

^{51}V NMR spectra

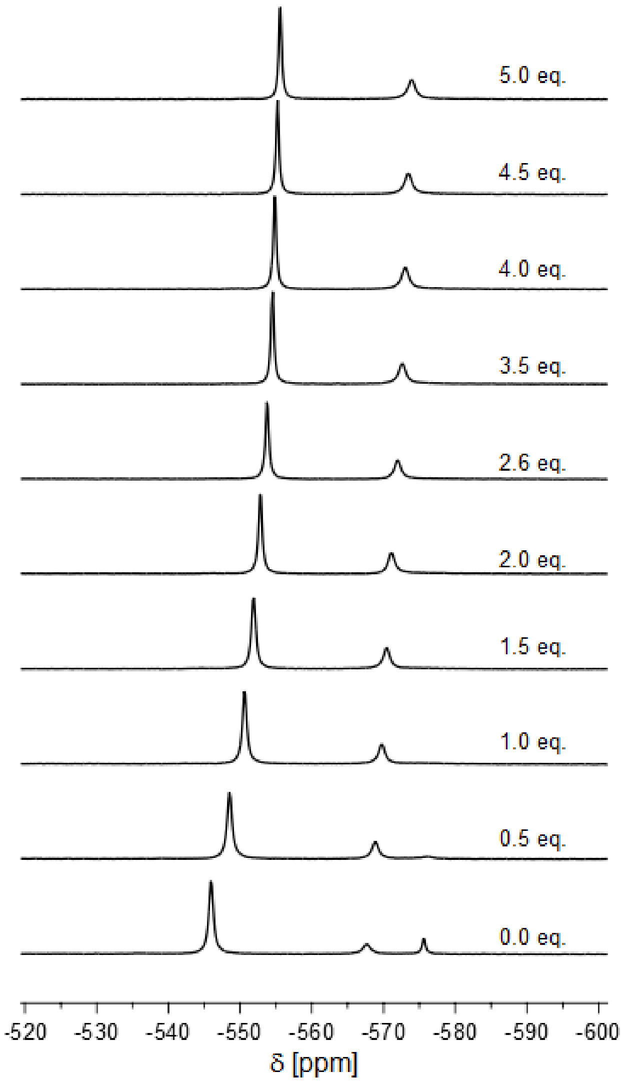

Figure ESI.1: ^{51}V NMR spectra of EPPS buffered solution (pH ~8.5} [100 mM EPPS; 1.5 mM $[Na_3VO_4]_t$; 25 °C].

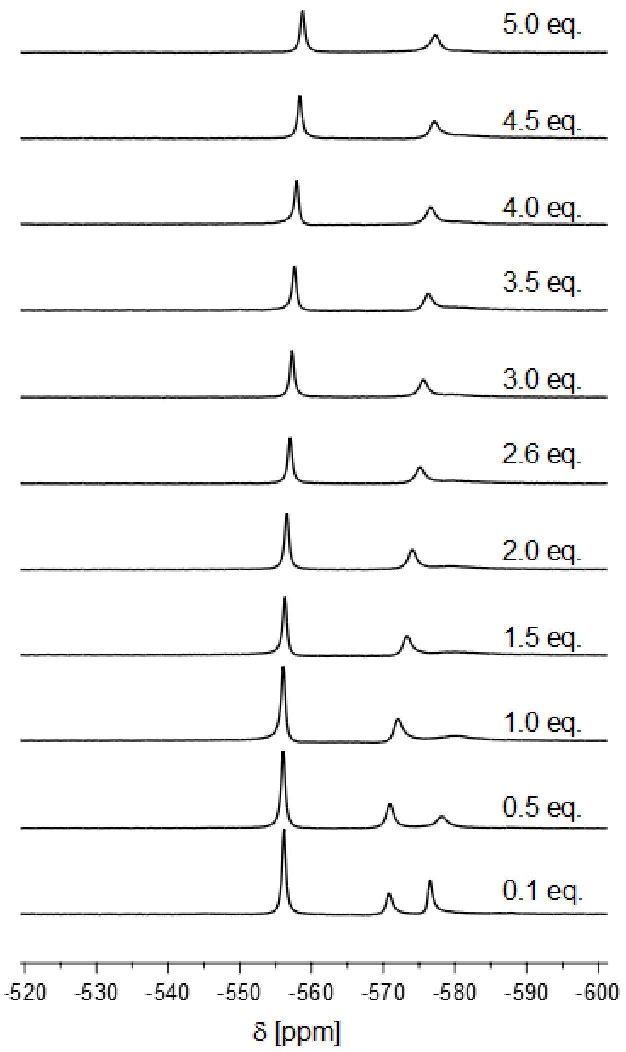

Figure S2: ^{51}V NMR spectra of HEPES buffered solution (pH ~7.6} [100 mM HEPES; 1.5 mM $[Na_3VO_4]_t$; 25 °C].

Fits to experimental ^{51}V NMR data

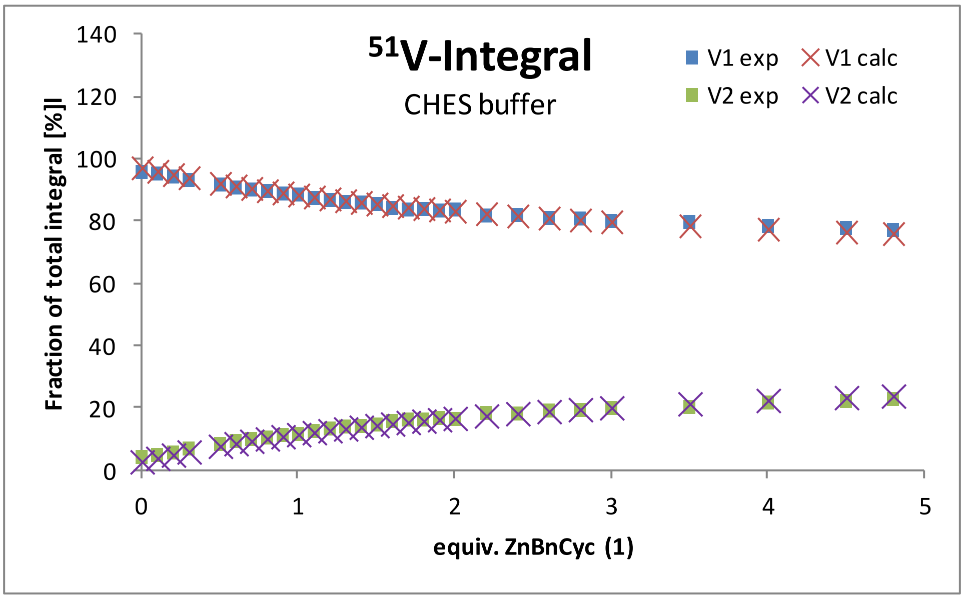

Figure S3: Fit to the ^{51}V NMR data of the titration in CHES buffered solution.

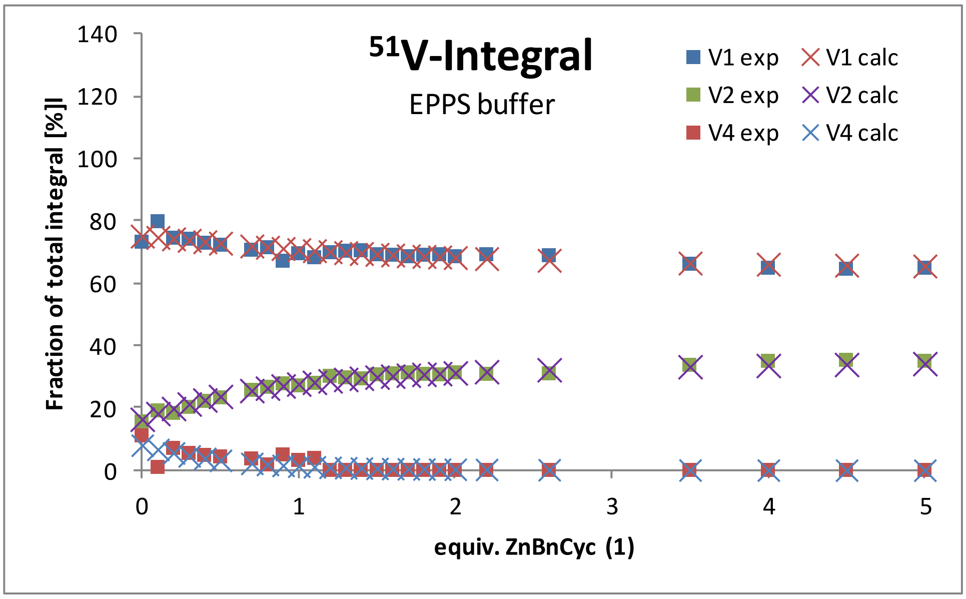

Figure S4: Fit to the ^{51}V NMR data of the titration in EPPS buffered solution.

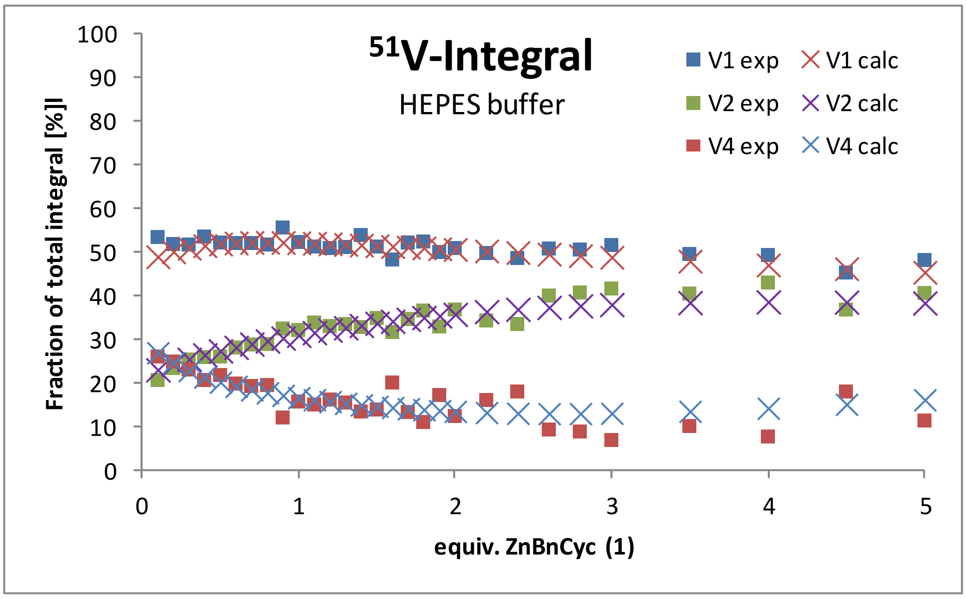

Figure S5: Fit to the ^{51}V NMR data of the titration in HEPES buffered solution.
